# Conservative Surgical Treatment of Bisphosphonate-Related Osteonecrosis of the Jaw with Er,Cr:YSGG Laser and Platelet-Rich Plasma: A Longitudinal Study

**DOI:** 10.1155/2018/3982540

**Published:** 2018-08-19

**Authors:** Rodolfo Mauceri, Vera Panzarella, Laura Maniscalco, Alberto Bedogni, Maria Ester Licata, Antonino Albanese, Francesca Toia, Enzo Maria Giuseppe Cumbo, Giuseppina Mazzola, Olga Di Fede, Giuseppina Campisi

**Affiliations:** ^1^Department of Surgical, Oncological and Oral Sciences, Sector of Oral Medicine “V. Margiotta”, University of Palermo, Palermo, Italy; ^2^Department of Sensor-Neural and Motor Surgery, Sector of Oral Medicine and Dentistry for Patients with Special Needs, University Hospital “Paolo Giaccone”, Palermo, Italy; ^3^Unit of Maxillofacial Surgery, Department of Neuroscience (DNS), University of Padua, Padua, Italy; ^4^Unit of Transfusional Medicine, University Hospital “Paolo Giaccone”, Palermo, Italy

## Abstract

**Introduction:**

The management of bisphosphonate-related osteonecrosis of the jaw (BRONJ), with no evidence-based guidelines, remains controversial. We aimed to evaluate the efficiency of a conservative surgical treatment combining Er,Cr:YSGG laser and platelet-rich plasma (PRP) for the treatment of BRONJ in cancer patients.

**Methods:**

We performed a longitudinal cohort study. Inclusion criteria were (1) age ≥ 18 years; (2) cancer diagnosis; (3) treatment with NBP because of the underlying cancer.

**Results:**

We consecutively recruited ten patients diagnosed with BRONJ in stage I or II. These patients underwent a surgical laser-assisted therapy together with autologous PRP. At the latest follow-up at 12 months, clinical improvement was observed in eight patients. Registration Number is IRCT20180329039159N1.

**Conclusion:**

We could successfully manage the BRONJ utilizing this combined protocol to heal the 30% of surgically treated sites and to improve the 50% of patients' lesions clinically. Our findings suggest that a surgical approach combined with Er,Cr:YSGG laser and PRP benefit cancer patients with general health issues.

## 1. Introduction

Bisphosphonate-related osteonecrosis of the jaw (BRONJ) is a severe adverse reaction of bisphosphonates (BPs) treatment; it is a relative rare but potentially serious, painful, and debilitating complication that can significantly affect the quality of life of cancer patients [1, 2].

The optimal treatment of BRONJ remains controversial; but the main objectives in the treatment are to control infection, to slow the disease's progression, and to promote tissue healing [1–4].

The BRONJ treatments are classified into surgical and non-surgical options. The non-surgical treatments include the use of systemic antibiotic therapy and oral antiseptic rinses, variably combined with hyperbaric oxygen therapy, low-level laser therapy, and medical ozone applications [5–9]. The surgical treatments proposed in the literature are divided into conservative approaches such as bone debridement, sequestrectomy, or more aggressive therapies such as resections of affected bone and jawbone reconstruction, if indicated [4, 10–12].

In the past, the surgical treatments were reserved only for advanced stages of BRONJ; the Italian Society of Oral and Maxillofacial Surgery (SICMF) and the Italian Society of Oral Pathology and Medicine (SIPMO) in 2012 recommended conservative surgery in lesions belonging to stages I and II, as defined by both societies, that can provide resolution of acute infection and offers long-term of well-being for patients [10, 11, 13–17].

A few clinical studies utilizing Er,Cr:YSGG laser-assisted conservative surgery have showed promising results in BRONJ treatment [6, 10, 15, 18].

Utilizing the Er,Cr:YSGG laser, during the necrotic tissue removal, eliminates thermal effect in cutting areas and surrounding tissues and provides antibacterial and biostimulative effects to reduce post-operative pain and promotes the tissue healing. The laser acts through a cutting effect in a “contact free” way and avoids any friction, which normally delays the healing's process and causes the thermal and mechanical trauma. It applies microfractures and microexplosions to remove the mineralized tissue and vaporize water to allow the rapid removal of the tissue layers that saves bone surface from any contaminations. The Er,Cr:YSGG laser, in particular, owns all these features, allowing a complete healing for both soft and hard tissues [19–23].

Autologous platelet concentrates, such as platelet-rich plasma (PRP), are increasingly applied as a new approach to regenerate tissues in oral surgery as they release high quantities of growth factors, including platelet-derived growth factor (PDGF), vascular endothelial growth factors (VEGF), and transforming growth factor-b (TGF-b) [24–27]. PDGF plays a role in healing hard and soft tissues by stimulating mitogenesis, chemotaxis, and producing fibronectin. High VEGF in wound sockets improves the formation of bone matrix and stimulates the neoangiogenesis. TGF-b stimulates the fibroblast chemotaxis and produces fibronectin and collagen to repair connective tissues and regenerate bones [28–30]. Indeed, PRP accelerates epithelial wound healing, decreases tissue inflammation, improves the regeneration of bone and soft tissues, and promotes tissue vascularization. Considering these benefits, PRP would be effective in BRONJ patients in the way that releases growth factors and stimulates the bone healing and neoangiogenesis, which is usually suppressed by BPs [26, 31–37]. Moreover, PRP as an autologous product possesses biocompatibility and safety.

This study aimed to evaluate the effect on clinical healing of a combined treatment consisting of laser-assisted surgery and PRP in cancer patients affected by BRONJ.

## 2. Patients and Methods

### 2.1. Study Design

We performed a prospective cohort study on consecutive cancer patients followed at the Unit of Oral Medicine of the University Hospital “P. Giaccone” of Palermo. The Institutional Local Ethics Committee of the University Hospital “P. Giaccone” of Palermo approved the study in 2015.

Technical and surgical procedures were done in accordance with the Declaration of Helsinki revised in 2000. (Table 1). All participants signed the informed consent.

### 2.2. Entry and Exclusion Criteria

BRONJ was defined as exposed or non-exposed osteonecrosis of the mandible or maxilla [2]. Patients were eligible for the study if they had (1) age ≥ 18 years; (2) cancer; (3) treatment with BPs because of the underlying cancer. Patients were excluded from the study if they had (1): previous history of irradiation to the maxillofacial area; (2) neoplastic involvement of the jaws; (3) previous surgical treatment to the jaws; (4) poor general conditions.

We diagnosed BRONJ in all cases through a clinical-radiological approach combining clinical examination and Computed Tomography (CT) of the affected jaws. A radiologist with experience and a special interest in head and neck imaging assessed and reported CT scan, while the local clinical team was in charge of the final diagnosis.

### 2.3. Clinical Examination

At first visit, we collected the clinical, drug, and dental history of patients, which consisted of the following: (1) age; (2) sex; (3) reason for BPs usage; (4) BPs type; (5) duration of BPs treatment; (6) cumulative dose of BPs; (7) concurrent use of steroids; (8) history of chemotherapy; (9) concurrent use of antiangiogenics; (10) concomitant diseases; (11) risk factors for BRONJ (e.g. history of diabetes); (12) clinical features of BRONJ; and (13) patients' habits (e.g. smoking and oral hygiene). We classified lesions following SICMF-SIPMO clinical and radiological staging system of BRONJ [2, 5].

### 2.4. Surgical Treatment

All patients underwent perioperative pharmacological treatment based on the administration of ampicillin and sulbactam (pre-operative regimens: 1g i.m. 2xdaily starting 1 day pre-operatively; post-operative regimens: 1g i.m. 2xdaily for 7 days) and metronidazole (pre-operative regimens: 500 mg per os 3xdaily starting 1 day pre-operatively; post-operative regimens: 500 mg per os 3xdaily for 7 days). The use of antiseptic (chlorhexidine 0,2% mouthwashes 30 ml swished up to 60 seconds, 3x daily 7 days pre-operatively and 15 days post-operatively) and sodium-hyaluronate (local application 3x daily 10 days post-operatively) was also prescribed (Table 2); autologous PRP (Plateltex ACT System, Biomed, Modena, IT) and surgical therapy were then prepared. To prepare the PRP and to induce its gelation, the materials provided by the manufacturer were used and the provided instructions followed [38].

All surgical procedures were performed under local anesthesia using 3% mepivacaine hydrochloride without adrenaline.

**Figure 1:**
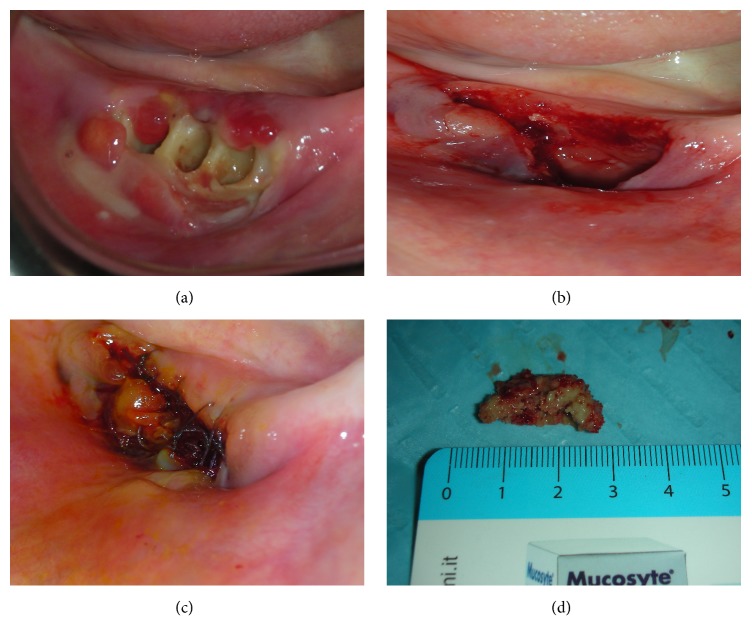
(a) Preoperative clinical view; (b) sutures; (c) postoperative view after sequestrectomy procedures; (d) bone fragment.

The surgical protocol consisted of elevation of a full-thickness mucoperiosteal flap to expose the surgical area; bony curettage (debridement) and sequestrectomy of the necrotic bone, whether required, using a Er,Cr:YSGG laser (Waterlase MD, Biolase Technology, San Clemente, CA, USA); application of autologous PRP; tension-free soft tissue closure (Figure 1).

The Er,Cr:YSGG laser permits photons with wavelength of 2.78 *μ*m and a pulsed duration of 140-200 microseconds with a repetition rate of 20 Hz. The laser device uses a pulsed energy source; a sapphire MS75 tip (Biolase, Inc.) with a length of 6 mm and diameter of 750 *μ*m was used with an 80% water and 40% air spray during irradiation. The sapphire tip was positioned 1 to 2 mm from the target tissue and was kept perpendicular to the irradiated bone surface. The power output of the laser can be varied from 0 to 6 W, while the beam spot size at the tip was 1.26·10 3 mm^2^.

### 2.5. Follow-Up

**Figure 2:**
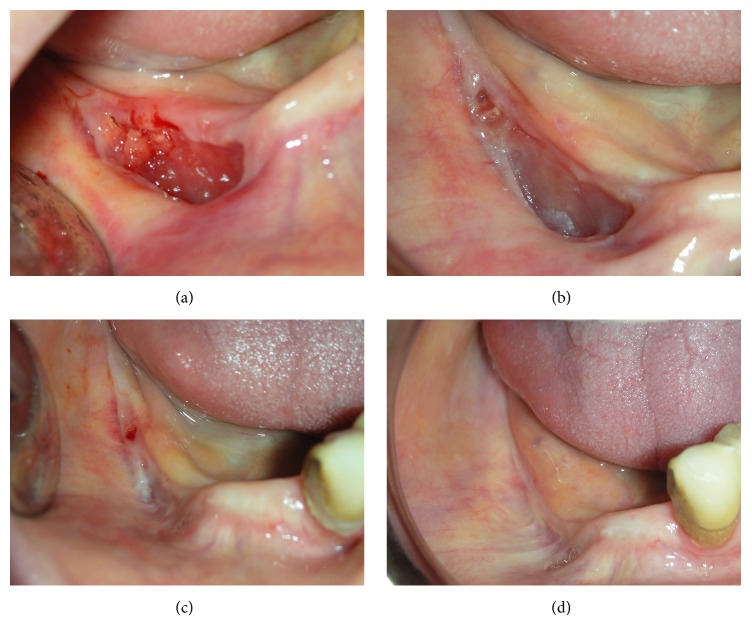
(a) Clinical view after 7 days; (b) after 1 month; (c) after 6 months; (d) after 12 months.

**Figure 3:**
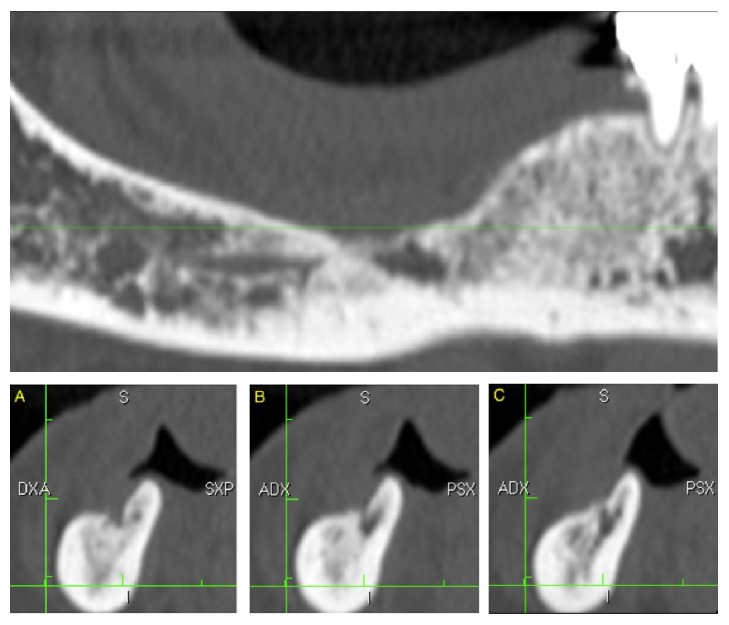
Radiologic outcome at 12 months' follow-up, CT scan slices.

We scheduled follow-up visits to remove the suture seven days after surgery and visited patients on the fifteenth day, and the visits were continued on months one, three, six, and twelve (Figures 2-3).

We performed Computer Tomography (CT) scans for all patients preoperatively and at the 12-month follow-up (T_1_) to restage the disease.

### 2.6. Main Outcome

We defined successful treatment as the absence of clinical and radiological signs of BRONJ relapse (healing) or, in turn, the transition from a higher stage to a lower one (improvement).

### 2.7. Statistical Analysis

Statistical units are the patients who satisfy the inclusion criteria of the study. Descriptive statistic was carried out. We summarized continuous variables with means and standard deviations and computed categorical variables frequencies distributions. We analyzed qualitative variables, staging and bone status, and compared them at baseline (T0) and after the treatment (T1). We applied the Wilcoxon signed-rank test with continuity correction for BRONJ staging. We analyzed all data using R software version 3.3.2. A p-value less than 0.05 was considered statistically significant.

## 3. Results

### 3.1. Patients' Features

During the study period, ten cancer patients completed the protocol and were available for the analysis. They were mostly females (70%), with a mean age 75,2 ± 5,94 years. Multiple myeloma was the most common diagnosis (40%), followed by breast (30%) and prostate cancer (30%).

All patients but one had been on monthly infusions of zoledronic acid (mean duration: 31,8 ± 25,76 months); this latter was shifted from zoledronic acid to ibandronic acid during treatment. Two patients (20%) were on systemic corticosteroid therapy and five had been exposed to chemotherapy; only one patient received both steroids and chemotherapy (Table 3).

Mandible was the most frequent site of BRONJ (90%). Eight patients showed frank bone exposure at the first visit (80%), while 20% did not and were classified as non-exposed BRONJ cases, based on the presence of oral pain and radiological signs of bone necrosis.

At baseline (T_0_), six patients were classified in stage IB (60%), two in stage IIA (20%), and the remaining two in stage IIB (20%) (Table 3), according to the SICMF-SIPMO clinical and radiological staging system of BRONJ [2].

### 3.2. Main Outcome

The wound healing was completed at the time of suture removal in 30% of patients. At the latest follow-up period (T_1_), we observed a clinical improvement in 80% of patients after surgical therapy that was confirmed by Wilcoxon test (p-value = 0.01187). In particular, six patients showed non-exposed bone (60%). Among them, three (30%) had no clinical and radiological signs of BRONJ (complete healing). Five patients showed a clinical improvement of symptoms (50%), while 2 did not show clinical improvement (20%). These latter had been preoperatively classified in stage IIA. Overall, at T_1_ four patients were in stage IA (40%) and three patients in IIA (30%). Results are shown in Table 4.

## 4. Discussion

We succeeded to manage BRONJ patients performing the conservative surgery treatment through Er,Cr:YSGG laser combined PRP, both able to enhance the bone and mucosal healing, with a successful outcome of 80% (30% patients with no clinical and radiological signs of BRONJ relapse and 50% with clinical improvement).

The use of laser technology for BRONJ treatment and its beneficial effects on tissue healing has been widely investigated in the last years. Many authors suggested the combination of low-level laser therapy (LLLT) with traditional surgical approach, to biostimulate the tissues healing [6, 39–41].

The use of Er,Cr:YSGG laser has a great potential in the hard tissues surgery; indeed Er,Cr:YSGG laser enables efficient resection of the maxilla without using conventional rotary instruments, as the laser produces a clear and precise cut with minimal injury to contiguous hard and soft tissues [6, 20, 23, 42, 43].

This device showed good results in conservative surgery approach of BRONJ treatment in different stages. Vescovi et al. reported several studies regarding the use of Er:YAG laser to treat BRONJ lesions, showing that Er:YAG seems to represent a high percentage of success, with significantly better results compared with the traditional surgical approaches [15, 39, 44, 45].

Many authors suggested the application of PRP to improve postsurgical wound healing. PRP gel stimulates the release of growth factors and promotes angiogenesis and bone and mucosal healing. In addition, PRP is autologous, biocompatible, and safe product [24–27, 29].

The properties of autologous platelet concentrates appear particularly useful in BRONJ surgical therapy, as the lack of vascularization represents one of the major factors on pathogenesis of BRONJ.

Coviello et al. reported a case series with seven patients taking BPs and affected by BRONJ referable to tooth extraction. They treated four of BRONJ patients by standard surgical debridement and sequestrectomy, while applying supplementary autologous PRP in the three. The authors observed wound healing's improvement and bone exposure reduction in the PRP group [46].

Martins et al. also studied the association of laser phototherapy (LPT) and PRP on healing outcome of BRONJ in cancer patients. These authors retrospectively compared the effects on wound healing of this protocol with a non-surgical (pharmacological therapy) and a surgical (pharmacological plus surgical therapy) one. They obtained higher rates of success, in terms of mucosal wound healing, in patients surgically treated with the LPT plus PRP protocol [47].

Longo et al. also achieved good results by studying the therapeutic effects in surgery therapy associated with PRP to promote BRONJ wounds healing [48]; performing a comparison with a surgical approach without PRP. They present higher success rate among patients treated with PRP (PRP group 93% of complete response versus control group 53% of complete response).

Lately, Kim et al. reported the application of leucocyte-rich and platelet-rich fibrin (L-PRF) in the treatment of BRONJ, with a complete resolution in 77% of cases treated and a delayed resolution in 18% of cases [49]. Comparing these results with ours, the success percentages are almost similar.

Notably, Mozzati et al. described the treatment of 32 BRONJ cases all belonging to stage IIB with the application of plasma rich in growth factors (PRGF) and reported a success rate of 100% with only temporary postoperative complications[24]. In 2017, also Maluf et al. reported two cases of medication related ONJ (classifiable as stage II) completely healed after a surgical treatment combined with the application of L-PRF[50].

Del Fabbro et al., in their systematic review, illustrated the data about fourteen studies published between 2007 and 2014; the BRONJ surgical treatment with an adjunct autologous platelet concentrates showed a satisfactory healing in 91,6% of the cases [36]. All these data are in agreement with our results in the treatment of BRONJ stage IIB.

Our study's limitations include the limited sample size and the presence of confounding factors, such as applied comedications (e.g. corticosteroids and/or different chemotherapy) in some patients. Indeed, these drugs could inhibit cell proliferation and with an antiangiogenic action could suppress vascular endothelial growth factor (VEGF) and fibroblast growth factor (FGF) [51–53].

## 5. Conclusion

Despite progress in the prevention of BRONJ, a specific treatment protocol to manage BRONJ is still missing.

Surgical removal of the necrotic bone should be performed with the Er,Cr:YSGG laser, that possesses remarkable properties with antibacterial and biostimulative effects, reduce postoperative pain and promote the tissue healing. Additionaly, PRP is an autologous product, biocompatible, easy to handle, and rich in growth factors and ameliorates the tissues healing in residual postsurgical wounds.

More prospective studies are needed to confirm this statement with a larger patients' sample. Considering the limitation of the present study, we could show that conservative surgical approach with Er,Cr:YSGG laser combined PRP benefits the management of early stages' BRONJ in cancer patients.

## Figures and Tables

**Table 1:** Technical procedures.

**Step**	**Procedure**
**1**	Clinical Evaluation

**2**	Computer Tomography (CT) evaluation Lesions' classification following SIPMO-SICMF staging system Scaling (when required) and oral hygiene instructions Pre-operatory medical therapy prescription (Table 2) Platelet rich-plasma (PRP) preparation

**3**	Surgical procedure (Figure 1) Er,Cr:YSGG Laser surgery PRP application Flap suture

**4**	Suture removal and clinical control

**5**	Follow-up visits at 15 days, one month, three, six, twelve months

**Table 2:** Prescribed medical therapy to enrolled patients.

**Operatory medical therapy**	
**Pre-**	Ampicillin and sulbactam: 1g i.m. 2xdaily starting 1 day before. Metronidazole: 500 mg per os 3x daily starting 1 day before. Chlorexidine 0,2% mouthwashes 30 ml swished up to 60 seconds, 3x daily 7 days before.

**Post-**	Ampicillin and sulbactam: 1g i.m. 2xdaily for 7 days. Metronidazole: 500 mg per os 3x daily for 7 days. Chlorhexidine 0,2% mouthwashes 30 ml swished up to 60 seconds, 3x daily 15 days post- operatively. Local application of Sodium-hyaluronate 3xdaily 10 days post-operatively.

**Table 3:** Descriptive statistics of the 10 enrolled patients.

Age (years)	75,2±5,94

Sex	

Male	3 (30%)

Female	7 (70%)

Smokers	2 (20%)

Cancer	

Multiple Myeloma	4 (40%)

Breast cancer	3 (30%)

Prostate cancer	3 (30%)

Comorbidities	

Diabetes	2 (20%)

Hypertensions	6 (60%)

Corticosteroids	2 (20%)

Osteoporosis	5 (50%)

Chemotherapy	5 (50%)

Rheumatoid arthritis	1 (10%)

Involved bone	

Maxilla	1 (10%)

Mandible	9 (90%)

BRONJ stage*∗*	

I A	0

I B	6 (60%)

II A	2 (20%)

II B	2 (20%)

Bone exposure	

Yes	8 (80%)

No	2 (20%)

Intravenous Bisphosphonates treatment time (mo)	31,8±25,76

*∗* BRONJ stage according to SICMF-SIPMO clinical and radiological staging system.

**Table 4:** Data of patients (Pt) at baseline (T_0_) and after the treatment (T_1_): multiple myeloma (MM); chemotherapy (CT), corticosteroids use (CST), and BRONJ stage according to SICMF-SIPMO staging system and presence of bone status (H= healed; R= reduction; NR= no reduction; E= exposed bone; NE= nonexposed bone).

**Pt**	**Sex**	**Age**	**Cancer**	**Bps**	**Bps duration (mo)**	**CT**	**CST**	**Affected Jaw**	**Stage T**_**0**_	**Stage T**_**1**_	**Bone Status T**_**0**_	**Bone Status T**_**1**_	**Output**
1	M	72	Prostate	Zoledronate	24	No	No	Upper	I B	I A	E	E	R

2	F	72	MM	Zoledronate	12	Yes	No	Lower	I B	I A	E	E	R

3	F	89	MM	Zoledronate	18	Yes	Yes	Lower	II B	H	E	NE	H

4	F	69	Breast	Zoledronate	36	Yes	No	Lower	I B	H	E	NE	H

5	F	80	MM	Zoledronate	60	Yes	No	Lower	I B	I A	NE	NE	R

6	F	74	Breast	Zoledronate	24	No	No	Lower	II B	II A	NE	NE	R

7	F	77	Breast	Zometa + Ibandronate	96	No	No	Lower	I B	H	E	NE	H

8	M	77	Prostate	Zoledronate	26	Yes	No	Lower	II A	II A	E	E	NR

9	F	70	MM	Zoledronate	18	No	No	Lower	I B	I A	E	NE	R

10	M	72	Prostate	Zoledronate	4	No	Yes	Lower	II A	II A	E	E	NR
